# Lossy mode resonance sensors based on lateral light incidence in nanocoated planar waveguides

**DOI:** 10.1038/s41598-019-45285-x

**Published:** 2019-06-20

**Authors:** Omar Fuentes, Ignacio Del Villar, Jesus M. Corres, Ignacio R. Matias

**Affiliations:** 1 Institute of Smart Cities, Public University of Navarre, 31006 Pamplona, Spain; 2 Department of Telecommunications and Electronics, Pinar del Río University, Pinar del Río, CP 20100 Cuba; 3 Department of Electrical and Electronic Engineering, Public University of Navarre, 31006 Pamplona, Spain

**Keywords:** Optical sensors, Optical metrology

## Abstract

The deposition of an indium oxide (In_2_O_3_) thin film on conventional planar waveguides (a coverslip and a glass slide) allows generating lossy mode resonances (LMR) by lateral incidence of light on the waveguide and by registering the optical spectrum in a spectrometer. This novel sensing system becomes an alternative to optical fibre, the substrate where LMR-based sensors have been developed so far, since it is easier to handle and more robust. An additional advantage is that cost effective waveguides, such as slides or coverslips, can be used in a platform that resembles surface plasmon resonance-based sensors in the Kretschmann configuration but without the need for a coupling prism and with the advantage of being able to generate TE and TM LMR resonances with metallic oxide or polymer thin films. The results are corroborated with simulations, which provide in-depth understanding of the phenomena involved in the sensing system. As a proof-of-concept for the optical platform, two refractometers were developed, one with low sensitivity and for a wide range of refractive indices, and the other with higher sensitivity but for a narrower refractive index range. The sensors presented here open up the path for the development of LMR-based chemical sensors, environmental sensors, biosensors, or even the generation of other optical phenomena with the deposition of multilayer structures, gratings or nanostructures, which is much easier in a planar waveguide than in an optical fibre.

## Introduction

In recent decades, progress has been achieved in the domain of sensors thanks to the ability to deposit thin films. One of the major milestones was achieved in 1982^1^ with the development of the first surface plasmon resonance sensor (SPR). The sensor was based on the utilization of the Kretschmann–Raether configuration^2^, which consists of an optical prism on which a metallic thin film coating is deposited. By introducing light with different angles of incidence, a surface plasmon polariton is excited in the metal–dielectric interface at a specific angle range, something that is also observed as a function of wavelength. Bloch surface waves can also be obtained with the Kretschmann configuration and be used for sensing^3^.

Another phenomenon, lossy mode resonance (LMR)^4,5^, can be observed with the same configuration^6^. However, LMR generation requires a thin film with different properties than those adequate for SPR generation. SPRs are obtained when the real part of the thin film permittivity is negative and higher in magnitude than both its own imaginary part and the permittivity of the material surrounding the thin film, whereas LMRs occur when the real part of the thin film permittivity is positive and higher in magnitude than both its own imaginary part and the material surrounding the thin film^4,7^. In view of the previous conditions, one would think that it is not possible to simultaneously observe both phenomena, SPR and LMR. However, there are materials, such as indium tin oxide (ITO), that present different properties depending on the operating wavelength range due to the material dispersion. Thanks to this property, it has been possible to obtain both an SPR and an LMR with the Kretschmann–Raether configuration and to compare their properties^6,8^.

Some important differences between SPRs and LMRs were observed. Unlike SPR, the position of LMRs in the optical spectrum depends directly on the coating thickness, which allows for simple tuning of the resonance wavelength^9^. In addition, the range of angles of incidence for excitation of LMRs is quite different from those adequate for SPR generation. SPRs are typically obtained for angles ranging between 40° and 70°^10,11^, whereas LMRs typically arise at near-grazing angle incidence, i.e. angles approaching 90°^5^. This explains why most of the experimental work on LMRs use optical fibre instead of the Kretschmann–Raether configuration^8,11–16^. With this last configuration, it is very difficult to impinge light at nearly 90°. However, though optical fibres show good characteristics, such as small size, immunity to electromagnetic interference (EMI), multiplexing and integration in a telecommunications network capability, wide range of operating temperatures, and remote sensing capacity due to their short diameter, there is a need for splicing the sensor head and they are affected by curvatures. Another important problem when using LMR-based optical fibre sensors is the polarisation. Though LMRs can be excited both with transverse electric (TE) and transverse magnetic (TM) polarised light, it is difficult to control the position of the resonance corresponding to TE polarisation and the resonance corresponding to TM polarisation. Depending on the nanocoating thickness and refractive index, the resonances either overlap with each other or, if they are separated, the full width at half maximum (FWHM) is not as small as it would be in the case of a polarising system, which reduces the resolution of the measurements^17,18^. So far, the best fibre optic platform is a D-shaped fibre^19,20^, which, thanks to its asymmetric cross-section, separates both TE and TM resonances with the aid of an in-line polariser and a polarisation controller that permit the control of polarisation in a standard single-mode fibre^21^ or by using a more sophisticated polarisation maintaining fibre^22^. Refractometers were fabricated using this type of platform, obtaining record sensitivities of 1.5 × 10^4^ nm/RIU in water and greater than 1 × 10^6^ nm/RIU in other higher refractive index media^15,21^ (to this purpose it is important to use substrates with a refractive index closer to the surrounding medium refractive index and metallic oxides with a high refractive index^4^). However, D-shaped optical fibres are expensive and controlling the polarisation is more difficult.

In this work, a planar waveguide was used as an alternative for optical fibre and the Kretschmann–Raether configuration. The setup consisted of the incidence of light by one of the lateral sides of a planar waveguide, which results in both sides of the waveguide being available for nanodeposition and hence for the generation of resonances. Light was collimated with the aid of multimode optical fibre, but even an LED source could be enough for achieving this goal. Between the light source and the planar waveguide, a polariser was used, which replaced the in-line polariser and the polarisation controller required for the optical fibre based setup (the polarisation controller must be tuned in every experiment, while the polarisation used in the setup proposed in this contribution can be easily positioned for TE or TM polarisation without a tuning that depends on the beating of the modes in the optical fibre).

The aim of the research presented in this paper was to demonstrate that LMRs for both TE and TM polarised light can be generated using this new platform. In addition, a refractive index sensor will be shown as an example of the application, where the wavelength shift and quality of the resonances while immersed in solutions of different refractive indices were similar to those obtained with the Kretschmann configuration and optical fibre, with the advantage of an easy-to-handle cost-effective setup that avoids the need for optical fibre splices. The reason for developing a refractometer is because the refractive index is the typical parameter analysed by researchers to assess the sensitivity and performance of an optical device^18^. However, the idea could be extended to almost any kind of sensor: biosensors, as in the previous citation, chemical sensors, and environmental sensors (gases, and volatile organic compounds). Finally, the experimental results were corroborated with numerical results obtained with FIMMWAVE, which assists in understanding the phenomena behind the experimental results.

## Results and Discussion

### Thin film and resonance characterization

**Figure 1:**
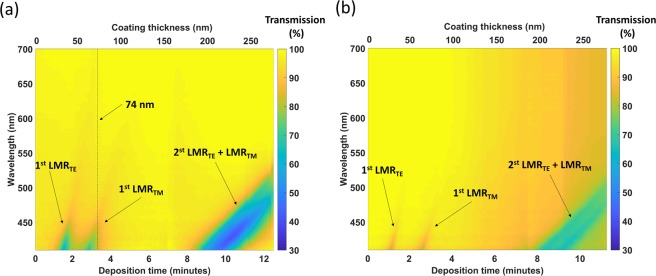
Optical spectrum evolution as a function of time (i.e. as the thickness of the thin film increases). The first TE and the first TM LMR were observed separately, while the second TE and TM LMR overlapped with each other. The following two planar waveguides were analysed: (**a**) coverslip and (**b**) glass slide. A video showing the evolution of the optical spectrum during the coverslip deposition is included in the Supporting Information: Visualization S1.

LMRs are generated due to the guidance of a mode in the nanocoating deposited on conventional soda lime glass waveguides (see Materials and Methods section). Soda lime glass presents a low absorbance in a wide wavelength range^23^, which allows monitoring over short wavelengths between 400 and 800 nm, using less expensive optical sources and detectors. After some deposition time, the first LMR was visible in the optical spectrum that was monitored, followed by the second one, and so on^17^. Figure 1 shows the evolution of the optical spectrum as a function of time while two different optical waveguides (a glass slide and a coverslip) were deposited with indium oxide (In_2_O_3_). No polarisation was introduced in the system during the deposition because the main interest was to see both TE and TM resonances. The first TE and TM LMRs were quite separated from each other, while for the second LMR both TE and TM resonances overlapped with each other. This phenomenon has also been observed in optical fibre and it depends on the refractive index of the thin film^15^. Moreover, a wider separation between TE and TM LMRs can be obtained by increasing the refractive index of the nanocoating.

The main difference observed by comparing the maps obtained in Fig. 1 was that for the coverslip the LMRs were more perceptible (the change in the transmission was higher). The explanation for this difference is that the width of the coverslip waveguide is 150 µm, whereas the width of the slide waveguide is 1000 µm. A thinner waveguide leads to a higher evanescent field for the waveguide modes^24^. This is the reason why the LMRs are more easily observed in Fig. 1a.

**Figure 2:**
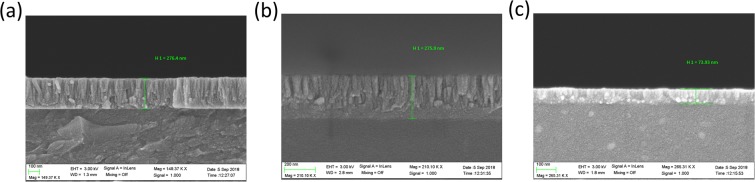
FESEM image of the cross-section of (**a**) a coverslip coated with a 276 nm In_2_O_3_ thin film (second LMR), (**b**) a slide coated with a 276 nm In_2_O_3_ thin film (second LMR), (**c**) a coverslip coated with 74 nm In_2_O_3_ thin film (first LMR).

The thin film thickness of both waveguides was measured with a field emission scanning electron microscope (model UltraPlus FESEM from Carl Zeiss Inc.) with an in-lens detector at 3 kV and an aperture diameter of 30 μm. The results presented in Fig. 2a,b reveal that the nanocoating has a thickness of 276 nm in both cases. In order to monitor the first LMR, a new deposition was performed on a coverslip waveguide. The deposition time was reduced to about 3 min and, this time, the nanocoating thickness was 74 nm (Fig. 2c).

### Characterisation of the first and second LMR

**Figure 3:**
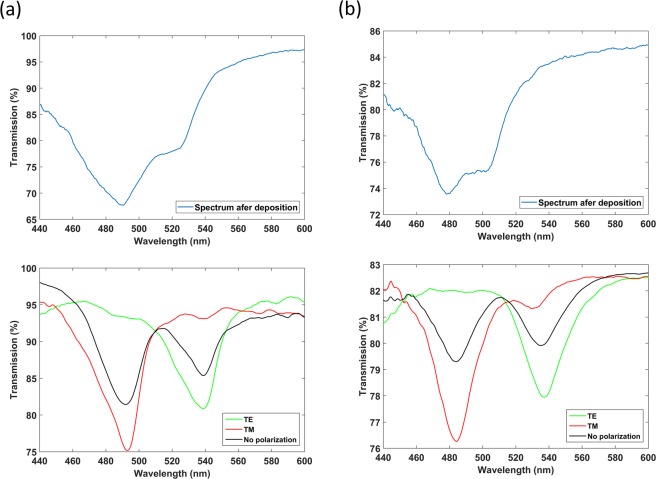
Optical spectrum after the deposition of an In_2_O_3_ thin film on a planar waveguide. The blue plot shows the spectrum in vacuum at the end of the deposition. The black plot shows the spectrum after the deposition in air. The green and red plots correspond to the spectrum obtained with TE and TM polarised light, respectively. The following two planar waveguides were analysed: (**a**) a coverslip and (**b**) glass slide.

Both the coverslip and the glass slide coated with a 276 nm thick In_2_O_3_ thin film (Fig. 2a,b) led to the generation of the second LMR, according to the colour map of Fig. 1. In Fig. 3, the optical spectra at the end of the deposition is presented for both waveguides under analysis. In both cases, a resonance can be observed centred at 480–500 nm. However, after the setup was extracted from the DC sputtering machine, the optical spectrum changed (black plots in Fig. 3). For both waveguides, there was a wavelength shift to the red and a reduction in the resonance depth. This was caused by the change from vacuum to air. The new optical spectrum reveals more clearly that the LMR is composed of the contribution of the TE LMR and the TM LMR (henceforward, we will call them LMR_TE_ and LMR_TM_). Indeed, if a polariser is introduced in the setup, both the LMR_TE_ and the LMR_TM_ can be obtained separately (green and red plot in Fig. 3). This simple process in this sensing setup is very complicated to achieve with optical fibre, which requires the use of specialty fibres, such as a D-shaped fibre, and the introduction of an in-line polariser and a polarisation controller in the experimental setup or a polarisation maintaining a D-shaped fibre^21,22^.

According to LMR theory, the second LMR is less sensitive than the first LMR^4,9^. This idea is confirmed in Fig. 1, where it is easy to observe that the wavelength shift as a function of thickness (slope) was slower for the second LMR compared to the first TE and TM LMR. This is the reason why the second LMR was used for a wider surrounding refractive index (SRI) range characterization without running the risk of the LMR shifting out of the wavelength range monitored in the experimental setup. Consequently, the optical spectrum was registered in air (SRI = 1) and in different solutions with refractive indices ranging from 1.333 to 1.508.

In addition, it is easier to observe that the coverslip is a better option in terms of visibility of the resonances in Fig. 3 than in Fig. 1. For the coverslip, a 15 and 20% power decrease is observed for TE and TM resonances, respectively (see Fig. 3a), whereas the depth of TE and TM resonances in the case of the slide is only 4 and 6%, respectively (see Fig. 3b). A similar proportion is observed in Figs S1 and S2 for the second lossy mode resonance, which reinforces this conclusion.

On the other hand, with the coverslip, which is thinner, the evanescent field of the modes is increased, as is the case for optical fibres, where it has been observed that deeper resonances are observed in fibres with reduced diameters compared to the same optical fibre without such a diameter reduction^25^. This is the reason why the losses in the lossy mode resonance region are higher with a coverslip, i.e. with a thinner waveguide. Regarding transmission values outside of this wavelength range, they are mainly due to absorption losses due to the material coating. Absorption losses should increase if the evanescent field of the modes is increased in a thinner waveguide. However, the coupling in a coverslip is more directive than in the slide because the light coming from the 200 µm fibre is better aligned with a 150 µm waveguide (the coverslip) than with a 1000 µm waveguide (the slide). In view of this, it is expected that light with a wider range of angles is transmitted in the slide, which implies that more reflections take place along the waveguide (if we see this from a geometric optics point of view). Consequently, the attenuation out of the lossy mode resonance wavelength range is higher in the case of the slide.

**Figure 4:**
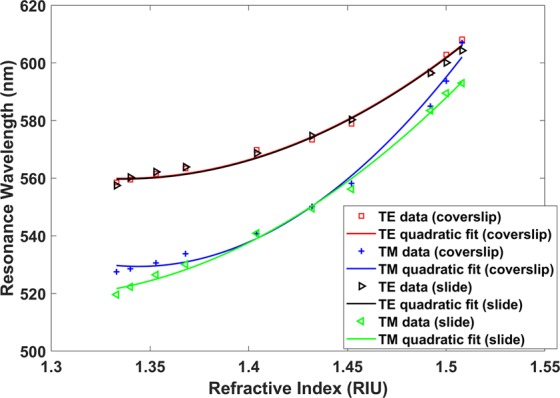
Representation of the central wavelength of the resonances as a function of the surrounding refractive index (SRI). In red and blue colour, TE data (coverslip) and TM data (coverslip) represent the wavelength shift of the second LMR_TE_ and the second LMR_TM_ respectively with a coverslip waveguide. In black and green colour, TE data (slide) and TM data (slide) represent the wavelength shift of the second LMR_TE_ and the second LMR_TM_ respectively with a glass slide waveguide.

The optical spectra corresponding to the second LMR_TE_ and the second LMR_TM_ are represented in Figs S1 and S2, whereas the wavelength shift of both LMR_TE_ and LMR_TM_ can be visualized in Fig. 4. Both coverslip and glass slide waveguides were used for the characterization, which allowed seeing that the depth on the resonance is different depending on the waveguide (Figs S1, S2**)**. This agrees with what was observed in Fig. 1, where the depth of the resonances obtained with the coverslip and the glass slide is different. Oppositely, the wavelength shift is very similar in both waveguides under analysis. This last question also agrees with Fig. 1, where the spectral shift was similar for the coverslip and the glass slide waveguide.

Another important conclusion is that the sensitivity is higher when the SRI increases, a phenomenon that is also observed in optical fibres^15^. As an example, for the coverslip the sensitivity in the range of 1.333–1.372 was 125 nm/RIU for TE polarisation and 162 nm/RIU for TM polarisation, whereas in the range 1.492–1508 the sensitivity was 1375 nm/RIU for TE polarisation and 731 nm/RIU for TM polarisation. This sensitivity increase for higher SRI values has also been visualized in LMRs obtained with optical fibres^9,15^.

Another interesting point that can be observed with the wide SRI range analysed is the different separation between LMR_TE_ and LMR_TM_ depending on the SRI. This behaviour is logical since the SRI is approaching the refractive index of the substrate. As a result, the thin film is surrounded by two media of similar refractive indices; the thin film waveguide asymmetry is very low and, hence, the performance at TE or TM polarisation is similar.

### Sensitivity optimization with the first LMR

As stated above, the sensitivity of the first LMR is higher than the sensitivity of the second LMR, an idea that is based on the wavelength shift of the resonances in Fig. 1 and on LMR theoretical and experimental results published with optical fibre waveguides^17^. However, the results presented in Fig. 1 show an important difficulty. The wavelength shift is faster, and it will be difficult to monitor a wide SRI range like that of Fig. 4.

On the basis of what was learned by monitoring the second LMR, the coverslip waveguide is a better candidate for monitoring the first LMR since the resonance is more visible and it is easier to stop the sputtering process adequately. That is why only the coverslip was analysed. In addition, as it was stated above, the sputtering process was stopped at about 3 min (see dotted vertical line in Fig. 1 for reference), and the thin film thickness was 74 nm after the sputtering process.

To have better knowledge of the LMR phenomenon in a planar waveguide, the experimental results were compared with theoretical results. For this purpose, FIMMWAVE software was used (details on the parameters used for the simulations and on the refractive index models used for the different elements are given in the Supporting Information).

**Figure 5:**
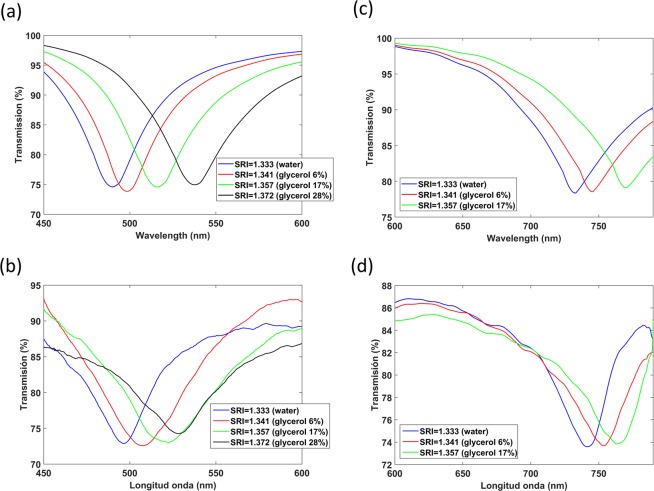
(**a**) Numerical and (**b**) experimental transmission spectra of the In_2_O_3_-coated coverslip (TM polarisation). (**c**) Numerical and (**d**) experimental transmission spectra of the In_2_O_3_-coated coverslip (TE polarisation).

Figure 5 shows results corresponding to the first LMR generated with a nanocoated coverslip waveguide. The results demonstrate that, according to Fig. 1, the LMR_TM_ and LMR_TE_ were obtained at different wavelength ranges, 490–540 nm and 730–780 nm, respectively. In addition, the most important conclusion is the sensitivity increase obtained with the first LMR. At TE polarisation, the sensitivity in the range of 1.333–1.357 was 929 nm/RIU, which means a 7-fold sensitivity increase compared to the sensitivity achieved with the second LMR_TE_. Regarding the first LMR_TM_, a sensitivity of 815 nm/RIU in the range of 1.333–1.372 was found, which means a 5-fold increase in sensitivity compared to the second LMR.

**Figure 6:**
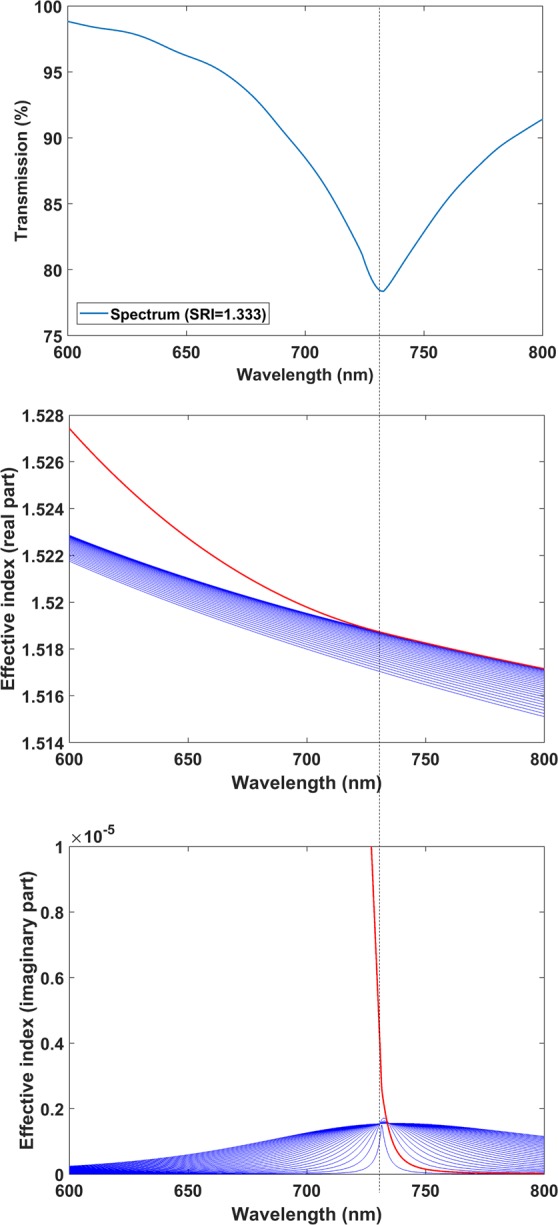
Optical spectrum of the first LMR_TE_ in water. The effective index of the 30 modes analysed is presented. One of the modes (red colour) experienced a transition to guidance in the thin film at the centre of the resonance.

In addition to the SRI analysis, to fully understand the LMR phenomenon in the coverslip waveguide, in Fig. 6 the LMR_TE_ in the range 600–800 nm is presented for the 74 nm coated coverslip waveguide with SRI = 1.333 (water). One of the modes experienced a transition to guidance in the thin film at the centre of the resonance (730 nm). This can be observed both in the real part and the imaginary part of the effective index of the modes. The rest of the modes showed a maximum in the imaginary part when this transition took place, which caused the reduction in the transmission around the transition wavelength.

The same analysis for TM polarisation is provided in the Supporting Information (Fig. S3) and similar conclusions can be extracted. Again the second mode of the coverslip waveguide experienced a transition to guidance in the thin film. The only difference is that, due to the different polarisation, the location of the resonance central wavelength and the transition to guidance of the mode was different (490 nm).

**Figure 7:**
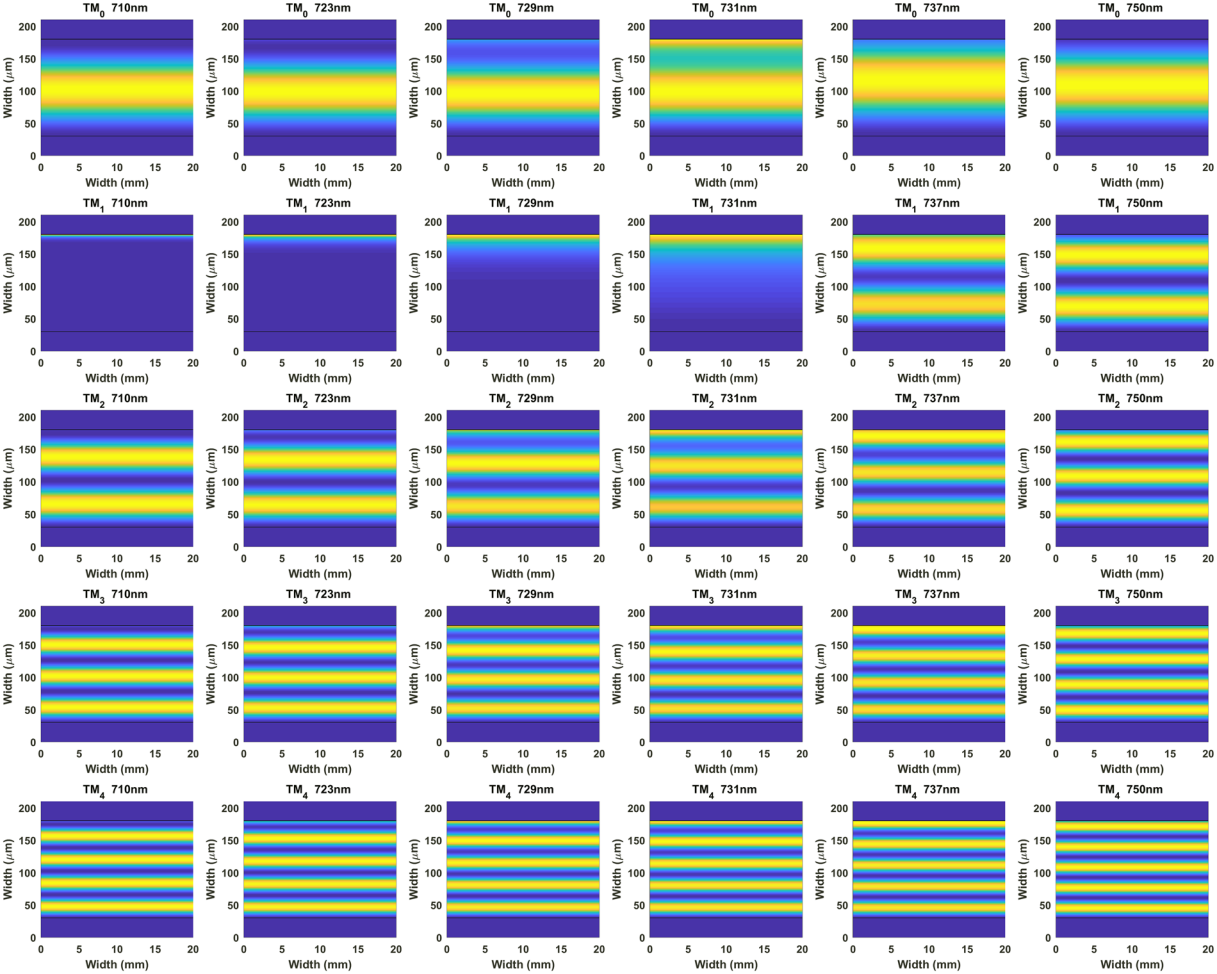
Optical field intensity distribution of TE_0_, TE_1_, TE_2_, TE_3_, and TE_4_ in the cross-section of a coverslip waveguide coated in the upper part with a 74 nm In_2_O_3_ thin film.

Finally, in Fig. 7 an analysis of the optical field intensity distribution for the first five coverslip modes is presented as follows: TE_0_, TE_1_, TE_2_, TE_3_, and TE_4_. All modes enhanced their evanescent field in the proximities of the LMR central wavelength, except for TE_1_, which experienced a transition to guidance in the thin film from 730 nm to shorter wavelengths until it concentrated all its optical field intensity in the thin film.

Again, the same analysis for TM polarisation is provided in the Supporting Information (Fig. S4), and similar conclusions can be extracted. Videos showing the evolution of the modes are also included in the Supporting Information: Visualization S2 for TE polarisation and Visualization S3 for TM polarisation.

## Conclusions

The lateral incidence in two In_2_O_3_ nanocoated conventional planar waveguides (a glass slide and a coverslip) has permitted the generation of LMRs and the ability to monitor them in the wavelength range 400–800 nm, where optical sources and detectors are cost-effective. On the basis of the results obtained previously with optical fibres instead of planar waveguides, the performance of this simple optical platform was improved by using a polariser, which allows for the separation of LMR_TE_ and LMR_TM_. Contrary to the domain of optical fibre sensors, where it is necessary to use costly devices, such as polarisation maintaining a D-shape or a D-shaped fibre combined with an in-line polariser and a polarisation controller, here it is only necessary to use a polarisation disk whose position can be easily switched to polarise TE and TM. The results presented here show that sensitivities of about 1000 nm/RIU in the 1.333–1.372 range can be obtained with the first LMR (about 5 to 7 times higher than that attained with the second LMR in the same refractive index range), whereas in the range 1.492–1.508 a sensitivity of 1375 nm/RIU with the second LMR can be obtained. It should be noted that the first LMR presents a higher sensitivity, but due to this higher sensitivity the LMR may shift out of the spectrum and, hence, the refractive index range that can be monitored with a single device is lower. The device presented here was not optimized towards the highest sensitivity. It is well-known that LMR sensitivity increases if the refractive index of the thin film is higher. Consequently, using refractive indices higher than that of In_2_O_3_ will improve the performance of the device. Moreover, it has also been observed that the sensitivity increases when the refractive index of the substrate is similar to that of the surrounding medium, leading to record sensitivities exceeding one million nanometers per refractive index unit^15^. Consequently, depending on the application it will be interesting to use waveguides of a material that is similar to the surrounding refractive index of the medium.

In view of the success of LMR-based optical fibre sensors in the domain of gas sensors, chemical sensors, or biosensors, optical fibres could be replaced with this easy-to-handle and robust setup that avoids the need for optical fibre splices. In addition, here it was proved by using conventional and low-cost slides and coverslips that it is possible to track LMRs at short wavelengths, where it is possible to use less expensive optical sources and detectors, but the system could be easily adapted to other needs. For instance, with borosilicate slides it could be possible to operate with a highly biocompatible system, while other materials could be easily applied for the setup presented here. Moreover, there is still a lot to do in terms of improving the sensitivity of LMR based sensors, and this sensitivity is related to the contrast between the thin film refractive index and the substrate, along with the similarity between the refractive index of the substrate and the refractive index of the surrounding medium^4^. The ability to use simple waveguides will widen the possibilities in terms of developing substrates with an adequate refractive index, at the same time higher refractive index thin films are explored towards sensitivities exceeding the record of more than 1 × 10^6^ nm/RIU obtained recently with tin oxide nanocoated D-shaped fibres^15^.

On the other hand, it is an interesting alternative to SPR sensors based on the Kretschmann configuration, because the devices presented here avoid the need for a coupling prism, thus allowing the deposition of thin films on both sides of the waveguide and the generation of two types of resonances, TE and TM. This multiplies the number of possibilities that this new sensing platform offers in terms of tracking the response to different materials and resonances. It may even be possible to obtain other optical phenomena with the deposition of gratings or nanostructures such as those explored in^26^, something that is much easier in a planar waveguide than in an optical fibre. The application of multilayer structures^27^ is another challenging possibility that could also be explored towards an increase in the quality factor and, hence, the figure of merit of the device. Moreover, with the setup presented here, it is possible to monitor the generation of the LMRs during the deposition process, which could be used for monitoring the thin film thickness if the refractive index of the material is known. In contrast, without knowing the refractive index, more complex processing would be needed that considers the wavelength separation between the TE and TM polarisation. In addition, the waveguide can be deposited on both sides, which can be used to obtain a double parameter sensor based on two different resonances or a dual channel microfluidic system could be implemented. Furthermore, the complexity of monitoring the deposition on both sides would increase; again, with the aid of a processing system, a new challenge could be to monitor the parallel or serial deposition of two materials on the waveguide. These are some examples that indicate the amount of work that remains to be done with this sensing platform.

Finally, the simplicity and low cost of this new platform, its versatility and potentiality in terms of using new structures and materials, and the possibility of the real-time detection of different species and compounds, etc., opens up the path for the development of new tools in many areas where the need for sensing is key, with very wide commercial and social repercussions.

## Materials and Methods

### Experimental setup

**Figure 8:**
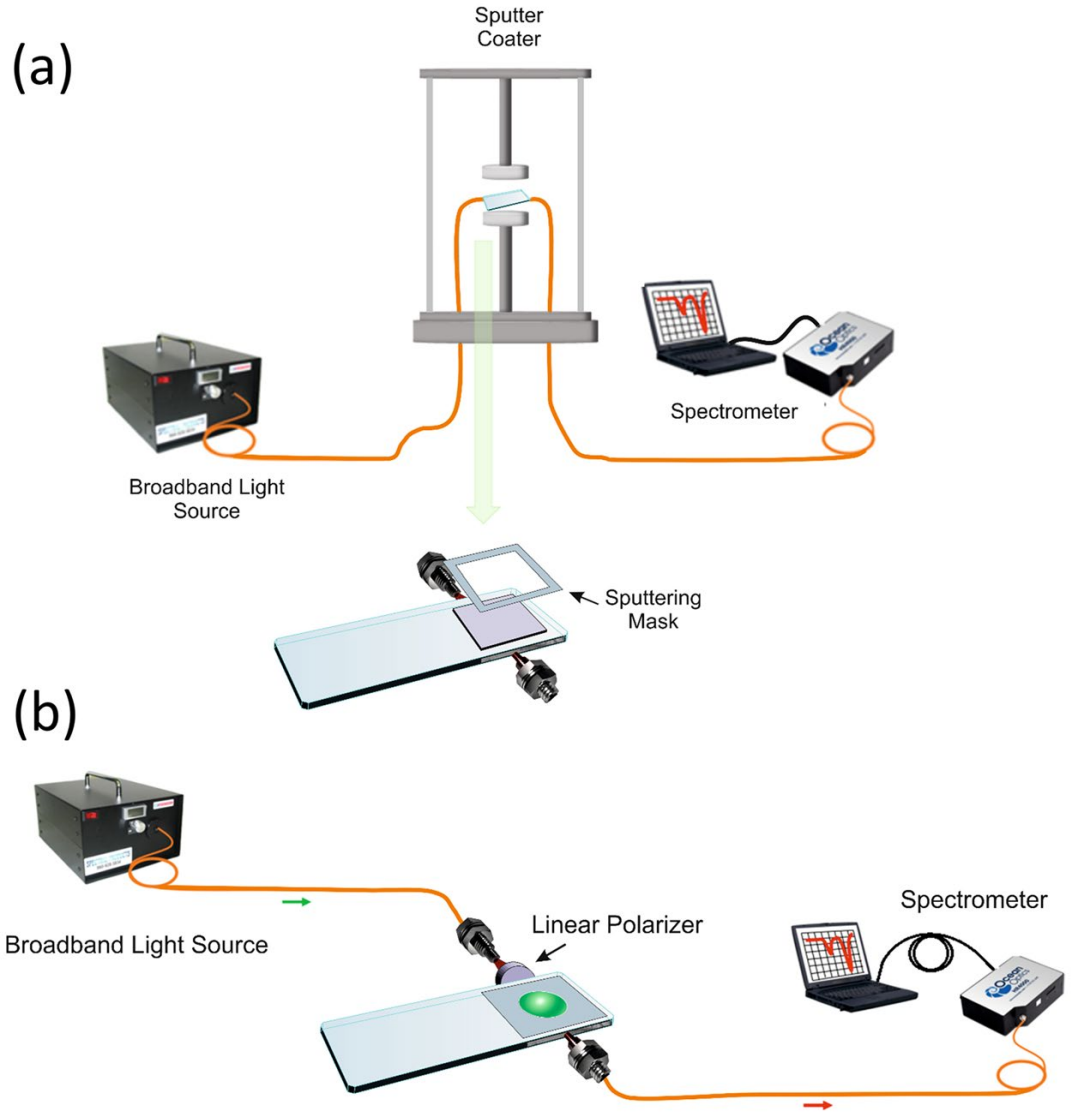
Setup used for the deposition and characterization of the utilization of the sensor as a refractometer. The system allows for the control of the polarisation of light and, hence, the excitation of both LMR_TE_ and LMR_TM_ resonances in the optical spectrum.

The experimental setup is described in Fig. 8. White light from an ASBN-W Tungsten-Halogen broadband source from Spectral Products Inc. (Putnam, FL, USA) was launched into a multimode optical fibre from Ocean Optics (200/225 µm of core/cladding diameter). This fibre was placed in front of one of the lateral sides of a planar waveguide and the output light was collected by another multimode optical fibre whose end was connected to an HR4000 spectrometer (OceanOptics Inc., Largo, FL, USA). As planar waveguides, we used RS France microscope slides (75 × 25 × 1 mm) and coverslips (18 × 18 × 0.15 mm), both made from soda lime glass, the most conventional material used for slides, although there is a wide range of materials that could be used, including borosilicate, the reference material for biocompatible applications. The planar waveguide was coated with In_2_O_3_ and put over a poly(methyl methacrylate) (PMMA) substrate material **(**Fig. 8**)**.

The thickness of the PMMA substrate was 5 mm with a refractive index slightly lower than the soda lime waveguides^28^. The purpose of the PMMA substrate was to support the fibres and the waveguide and to allow the orientation of the fibres to the lateral side of the waveguides.

The setup was placed in a DC sputtering machine (K675XD from Quorum Technologies, Ltd.). The parameters used in the experiment included an argon partial pressure of 8 × 10^−2^ mbar and an intensity 150 mA. The In_2_O_3_ sputtering target (57 mm in diameter and 3 mm in thickness) was purchased from ZhongNuo Advanced Material Technology Co. The glass waveguide was positioned at a distance of 7 cm from the target and the optical spectra was monitored continuously during the deposition process, which lasted 3 min for the first LMR and 9 min more for the second (i.e. 12 min). During the deposition process, a mask was used to avoid material deposition at the borders of the waveguide where the light from the fibres was introduced.

Before starting the deposition, the optical spectrum was taken as a reference signal. Then, each transmission spectrum was calculated by dividing the current spectrum by the reference signal during the deposition process. In this way, the colour map of Fig. 1 was obtained. After the deposition, a linear polariser LPVIS050 from Thorlabs was introduced between the output of the optical fibre that launched light in the waveguide and the waveguide itself. This excited the waveguide with linearly polarised light, which can be oriented horizontally or vertically (i.e. the electric field is oriented horizontally or vertically). This allowed for the separation of LMR_TE_ or LMR_TM_, which are deeper and easier to track when the device is used as a sensor. Consequently, the polariser was used for the characterization of the devices as a function of refractive index (more explanations and a schematic of the waveguide excitation with horizontal or vertical polarisation is given in the Supplementary Information, Fig. S5).

### Refractometer characterization

Once the waveguides were deposited with In_2_O_3_ and a polariser was introduced to separately obtain LMR_TE_ or LMR_TM_, the waveguides were tested as refractometers by pouring liquids of different refractive indices on the thin film coated waveguide.

As in the deposition process, a reference signal was taken. However, this time a non-deposited waveguide was used to obtain the reference signal and, after that, the non-deposited waveguide was replaced with the deposited one and the sensor signal was taken. In addition, the resulting spectra were then processed with an algorithm in Matlab® to obtain the corresponding peak wavelengths of Fig. 4.

To perform the measurements, the coated region was sequentially submerged in several glycerine solutions (Panreac® Technical Grade). All solutions were carefully prepared and stirred for several hours. Then, their refractive indices at a wavelength of 589 nm were measured with a commercial refractometer (Mettler Toledo® Refracto 30GS) with an accuracy of 0.0005. The refractive indices for water, and 6%, 17% and 28% glycercol in water were 1.333, 1.341, 1.357, and 1.372, respectively. For the simulations, the dispersion of the refractive index of water and glycerine according to^29,30^ was considered. To obtain a wider set of refractive indices like those represented in Fig. 4, the following Cargille refract index liquids Series A and Series AA were also used: 1.404, 1.432, 1.452, 1.492, 1.5, and 1.508.

## Supplementary information

Supporting information

Visualization S1

Visualization S2

Visualization S3
