# Author Correction: Lossy mode resonance sensors based on lateral light incidence in nanocoated planar waveguides

**DOI:** 10.1038/s41598-020-61551-9

**Published:** 2020-03-06

**Authors:** Omar Fuentes, Ignacio Del Villar, Jesus M. Corres, Ignacio R. Matias

**Affiliations:** 1 Institute of Smart Cities, Public University of Navarre, 31006 Pamplona, Spain; 2 Department of Telecommunications and Electronics, Pinar del Río University, Pinar del Río, CP 20100 Cuba; 3 Department of Electrical and Electronic Engineering, Public University of Navarre, 31006 Pamplona, Spain

Correction to: *Scientific Reports* 10.1038/s41598-019-45285-x, published online 20 June 2019

Three supplementary files containing Visualizations S1-S3 were omitted from the original version of this Article. This has been corrected in the HTML version of the Article; the PDF version was correct at the time of publication.

