# Supplementary material for: Lossy mode resonance sensors based on lateral light incidence in nanocoated planar waveguides
Source: Sci Rep. 2019 Jun 20;9:8882. doi: 10.1038/s41598-019-45285-x (PMC6586632; doi:10.1038/s41598-019-45285-x)
Supplement: Supplementary file 1 — Supporting information [file 41598_2019_45285_MOESM1_ESM.docx]

SUPPORTING INFORMATION to the manuscript

Lossy mode resonance sensors based on lateral light incidence in nanocoated planar waveguides

Omar Fuentes^1,2^, Ignacio Del Villar^1,3^*, Jesus Corres^3^, Ignacio R. Matias^1^

^1^Institute of Smart Cities, Public University of Navarre, 31006 Pamplona, Spain

^2^Department of Telecommunications and Electronics, Pinar del Río University, Pinar del Río CP 20100, Cuba

^3^Department of Electrical and Electronic Engineering, Public University of Navarre, 31006 Pamplona, Spain

[*Ignacio.delvillar@unavarra.es](mailto:*Ignacio.delvillar@unavarra.es)

Characterization of the Second LMR

The evolution of the spectrum for the 276 nm thick In_2_O_3_ coated coverslip and glass slides as a function of SRI can be observed in **Figure S1** (TE polarisation) and in **Figure S2** (TM polarisation). It is easy to observe the sensitivity increase at higher SRI values, according to the results of **Figure 4**. Regarding the depth of the attenuation bands, there was a progressive reduction in this parameter as a function of the SRI. This can be explained because for this refractive index the evanescent field of the modes increase (the optical losses increase) and the reference signal has been taken in air at the beginning of the experiment. If a new reference was taken any time a new refractive index was measured, this effect would be mitigated, but for the sake of simplicity and since it is possible to track the resonance, this method has not been applied. Finally, it is important to highlight that, according to **Figure 3**, the application of a slide waveguide leads to the generation of LMRs whose attenuation depth is not as notorious as that attained with coverslips. This indicates that coverslips are more adequate for LMR generation than glass slides and, in general, a thin waveguide is desired for the optical setup proposed in this work.


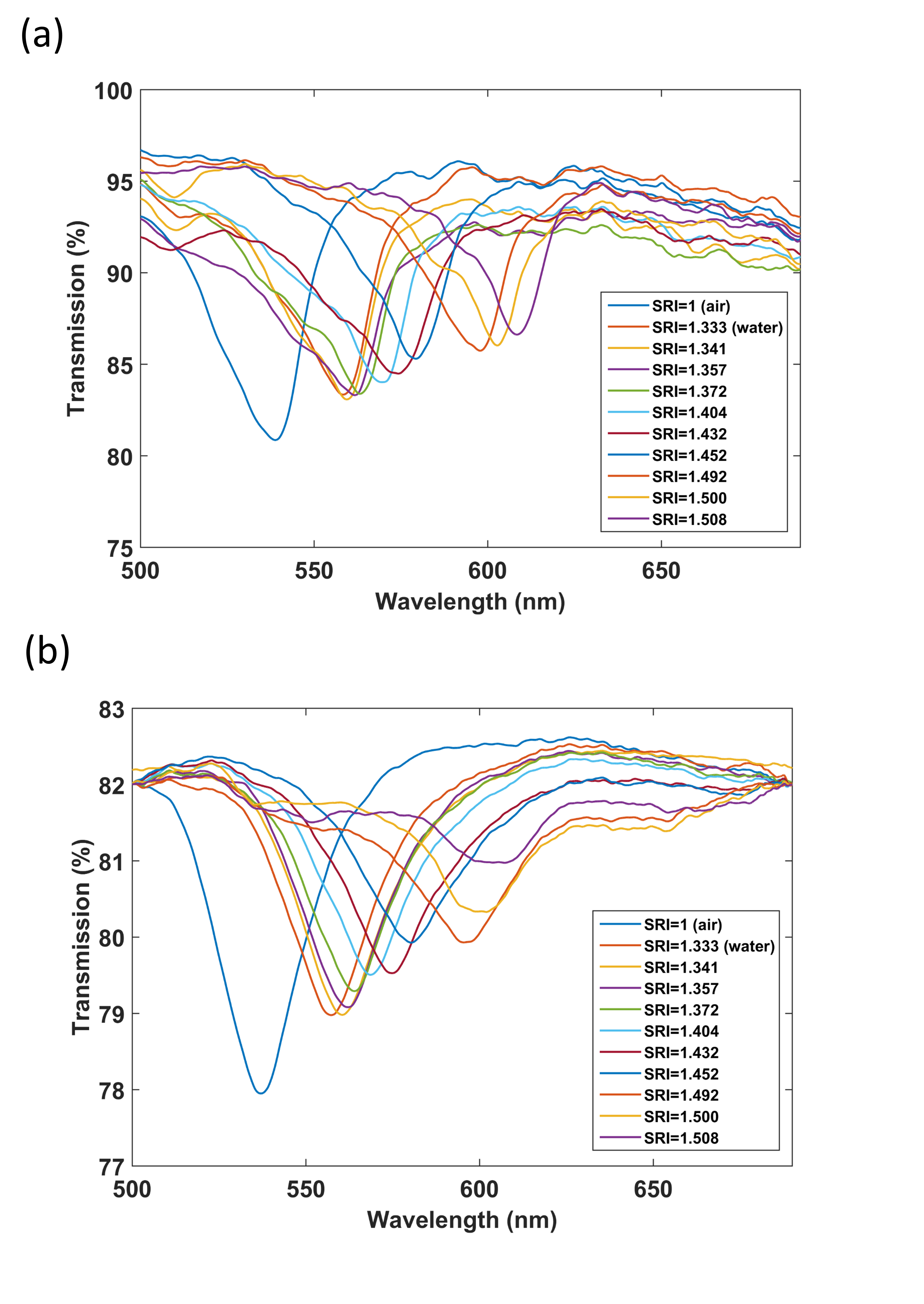


**Figure S1.** Transmission spectra of In_2_O_3_-coated waveguides at TE polarisation for a) a coverslip and b) a glass slide.


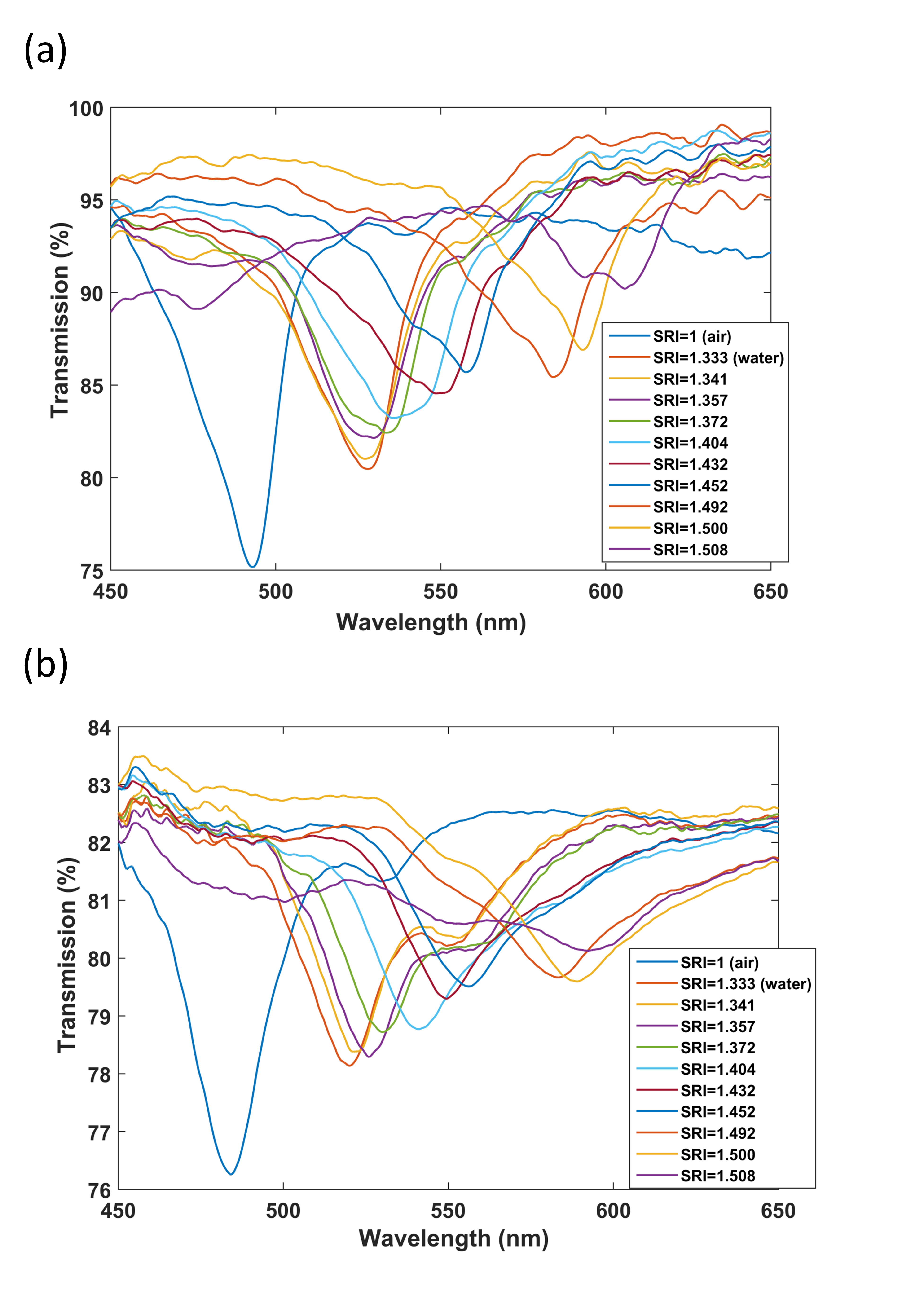


**Figure S2.** Transmission spectra of In_2_O_3_-coated waveguides at TM polarisation for a) a coverslip and b) a glass slide.

FIMMWAVE Simulations

Propagation through the coverslip waveguide was obtained with FIMMPROP, an integrated module of FIMMWAVE. The finite difference method (FDM) with the Quasi 2D version was used to calculate the modes and the fields in the cross section of the waveguide for a total number of 30 modes, which provided good convergence in the results. In addition, a Gaussian source of 200 µm half width at half maximum was used according to the 200 µm multimode fibre used in the experiments for exciting the planar waveguide.

Regarding the refractive index of the waveguide, since both the microscope slides and the coverslips were made of soda lime glass, the refractive index model of ^1^ was used. The planar waveguide was placed on a poly(methyl methacrylate) (PMMA) substrate material and the refractive index of this material was modelled according to ^2^. In addition, in view that the final application was a refractometer, the surrounding refractive index analysed was water, according to the model of ^3^. Finally, by considering the experimental position in water of LMR_TE_ and LMR_TM_ for the 74 nm In_2_O_3_ coated coverslip waveguide, the refractive index for In_2_O_3_ that best fitted the experiments was 1.82+0.0015i, a value that is located in the range 1.81–1.91 indicated in ^4^ at a wavelength of 550 nm. Finally, the surrounding medium and the PMMA layer were considered as infinite media.

The results of **Figure S3** complement those of **Figure 6**, where LMR_TE_ in the range 600–800 nm was presented for the 74 nm coated coverslip waveguide with SRI=1.333 (water). Conversely, LMR_TM_ for the same conditions in **Figure S3** is located in the range 400–600 nm. As for the case of TE, here one of the modes experienced a transition to guidance in the thin film at the centre of the resonance at 480 nm. This can be observed both in the real part and the imaginary part of the effective index of the modes. The rest of the modes showed a maximum in the imaginary part when this transition took place, which caused the reduction in the transmission around the transition wavelength. In general, after analysing the transition of a mode at TE and at TM polarisation, the main conclusion is that both transitions are similar, with the exception that the location of the transition is different due to the asymmetry of the refractive index between the two media surrounding the In_2_O_3_ thin film.

In addition to the analysis of the effective index of modes, the optical field intensity distribution of the first five coverslip TM modes is also presented in **Figure S4** as follows: TM_0_, TM_1_, TM_2_, TM_3_, and TM_4_. All modes enhanced their evanescent field in the proximities of the LMR central wavelength, except for TM_1_, which experienced a transition to guidance in the thin film from 480 nm to shorter wavelengths until it concentrated all of its optical field intensity in the thin film. This mode order, number 1, coincides with the TE_1_ mode guided at TE polarisation. However, this is not always the case. In principle, as the imaginary part or the thickness of the nanocoating increases higher order modes are guided in the thin film ^5^.


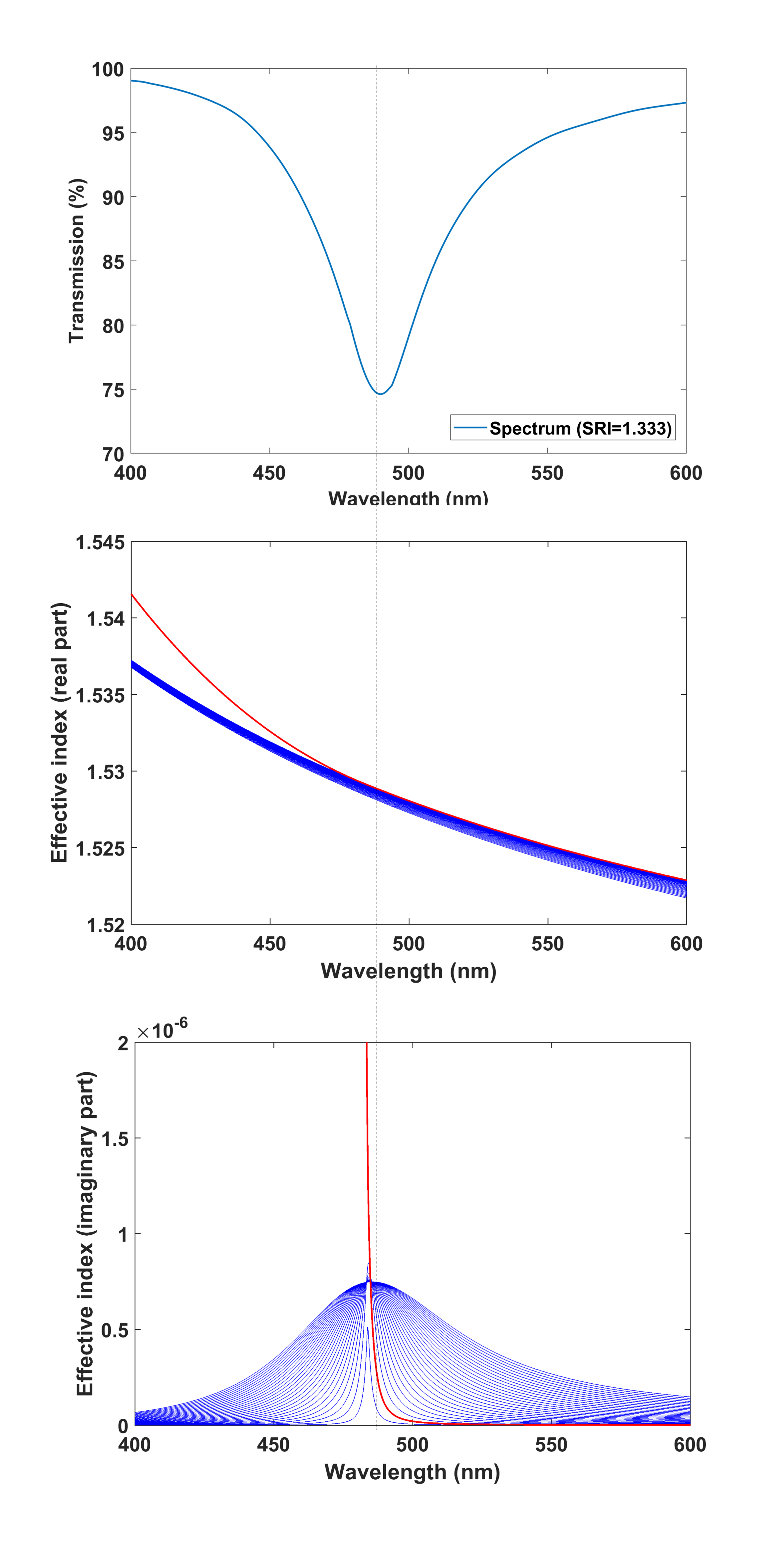


**Figure S3.** Optical spectrum of the first LMR_TM_ in water. The effective index of the 30 modes analysed is presented. One of the modes (red colour) experienced a transition to guidance in the thin film at the centre of the resonance.


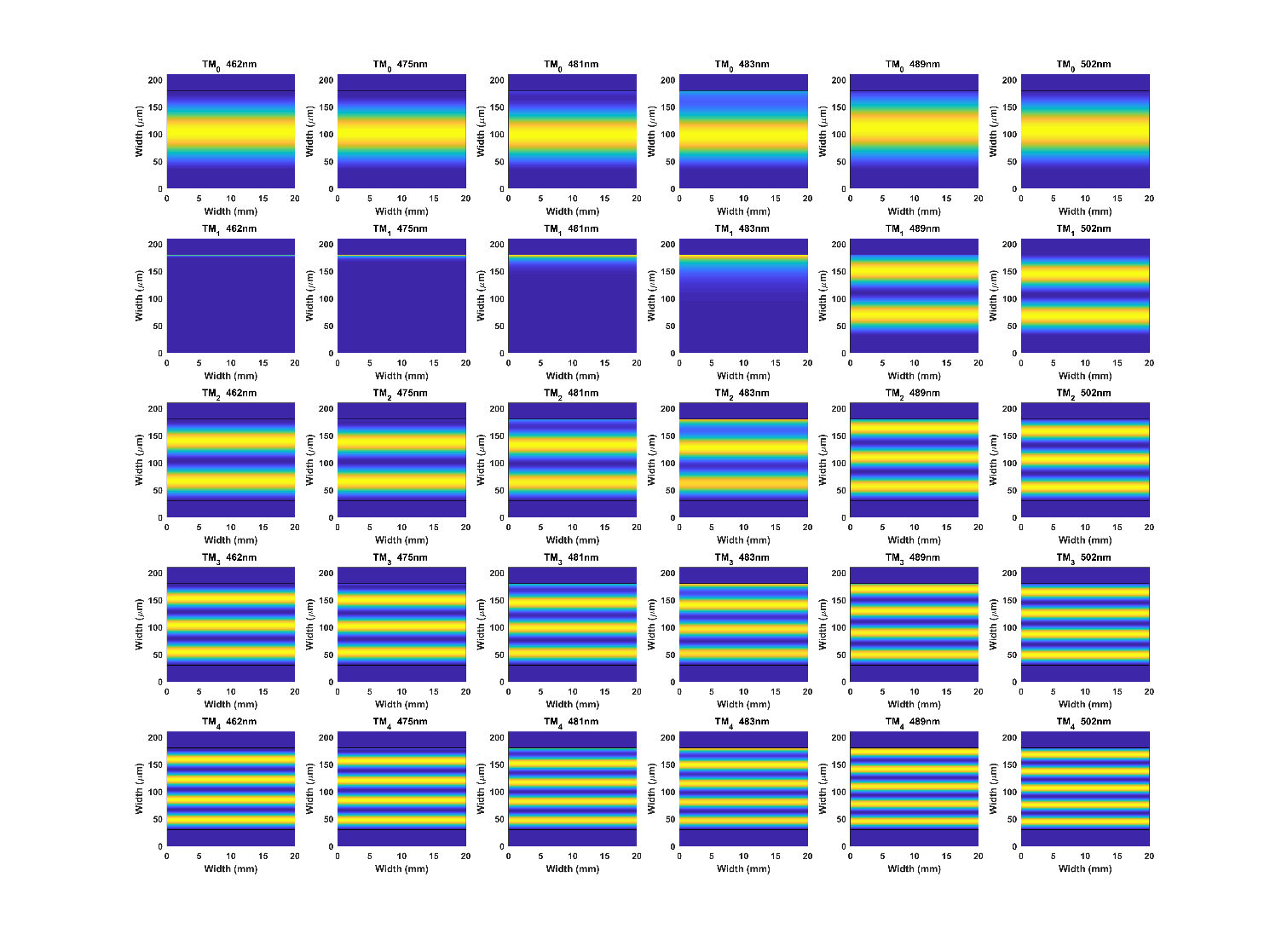


**Figure S4.** Optical field intensity distribution of TM_0_, TM_1_, TM_2_, TM_3_, and TM_4_ in the cross-section of a coverslip waveguide coated in the upper part with a 74 nm In_2_O_3_ thin film.

Vertical and Horizontal Polarisation towards Excitation of TM and TE LMRs

As stated in the Materials and Methods section, light is polarised with the aid of a linear polariser **(Figure S5)**. Assuming that a plane wave is transmitted, by rotating the polariser it is possible to control the orientation of the electric field. If the polarisation is horizontal (the electric field E is oriented horizontally and the magnetic field H vertically), the electric field is transversal to the plane of incidence (TE) and only TE modes are excited. Consequently, LMR_TE_ is obtained. However, if the polarisation is vertical (the electric field E is oriented vertically and the magnetic field H horizontally), the magnetic field H is transversal to the plane of incidence (TM) and only TM modes are excited. As a result, LMR_TM_ is generated. The different position of both resonances obeys to the different boundary conditions at the interface between the waveguide and the thin film and at the interface between the thin film and the surrounding medium, which leads to the well-known Fresnell reflection coefficients, which differ from each other depending on whether the TE or TM case is being addressed. This is why the global reflectivity is different in both cases, as proven in ^6^.

**Figure S5.** Schematic view of LMR excitation for: (a) Horizontally polarised light (LMR_TE_ is excited); (b) Vertically polarised light (LMR_TM_ is excited).

References

1. Rubin, M. Optical properties of soda lime silica glasses. *Sol. Energy Mater.* **12,** 275–288 (1985).

2. Sultanova, N., Kasarova, S. & Nikolov, I. Dispersion Properties of Optical Polymers. in *ACTA PHYSICA POLONICA A* **116,** 585–587 (2009).

3. Daimon, M. & Masumura, A. Measurement of the refractive index of distilled water from the near-infrared region to the ultraviolet region. *Appl. Opt.* **46,** 3811–3820 (2007).

4. Senthilkumar, V. & Vickraman, P. Annealing temperature dependent on structural, optical and electrical properties of indium oxide thin films deposited by electron beam evaporation method. *Curr. Appl. Phys.* **10,** 880–885 (2010).

5. Del Villar, I., Matias, I. R., Member, S., Arregui, F. J. & Achaerandio, M. Nanodeposition of Materials With Complex Refractive Index in Long-Period Fiber Gratings. *Lightwave* **23,** 4192–4199 (2005).

6. Homola, J. *Surface Plasmon Resonance Based Sensors*. (Springer, 2006).
